# A Multi-Layer Classification Approach for Intrusion Detection in IoT Networks Based on Deep Learning

**DOI:** 10.3390/s21092987

**Published:** 2021-04-24

**Authors:** Raneem Qaddoura, Ala’ M. Al-Zoubi, Hossam Faris, Iman Almomani

**Affiliations:** 1 Information Technology, Philadelphia University, Amman 19392, Jordan; rqaddoura@philadelphia.edu.jo; 2 School of Science, Technology and Engineering, University of Granada, 18010 Granada, Spain; ala.m.zoubi@gmail.com or; 3 King Abdullah II School for Information Technology, The University of Jordan, Amman 11942, Jordan; i.momani@ju.edu.jo or; 4 School of Computing and Informatics, Al Hussein Technical University, Amman 11831, Jordan; 5 Security Engineering Lab, Computer Science Department, Prince Sultan University, Riyadh 11586, Saudi Arabia

**Keywords:** intrusion detection, classification, neural network, deep learning, oversampling, SMOTE, imbalanced, IoTID20

## Abstract

The security of IoT networks is an important concern to researchers and business owners, which is taken into careful consideration due to its direct impact on the availability of the services offered by IoT devices and the privacy of the users connected with the network. An intrusion detection system ensures the security of the network and detects malicious activities attacking the network. In this study, a deep multi-layer classification approach for intrusion detection is proposed combining two stages of detection of the existence of an intrusion and the type of intrusion, along with an oversampling technique to ensure better quality of the classification results. Extensive experiments are made for different settings of the first stage and the second stage in addition to two different strategies for the oversampling technique. The experiments show that the best settings of the proposed approach include oversampling by the intrusion type identification label (ITI), 150 neurons for the Single-hidden Layer Feed-forward Neural Network (SLFN), and 2 layers and 150 neurons for LSTM. The results are compared to well-known classification techniques, which shows that the proposed technique outperforms the others in terms of the G-mean having the value of 78% compared to 75% for KNN and less than 50% for the other techniques.

## 1. Introduction

Internet of Things (IoT) is a novel innovation that emerged in recent years as the new big thing [1]. IoT can be described as a system of interconnected objects of mechanical and digital machines, computing devices, and living creatures such as animals and people that are provided with unique identifiers (UIDs) in order to transfer data over a network [1,2]. This operation usually occurs without the need for human-to-computer or human-to-human interactions [3].

A thing in the IoT can be, for example, a heart monitor implant for a person, a biochip transponder (lightweight sensors) in an animal farm, a web-connected sensor that improves the manufacturing process, or a built-in sensor to inform the current status and maintenance needs of cars that own an Internet Protocol (IP) address to be able to connect over the Internet [4].

This technology offers organizations and companies huge business value in fields such as industry, health, agriculture, education, and tourism [5]. Therefore, it provides opportunities to several parties (consumers, companies, and governments) to benefit from this technology’s features and advantages. However, most of the IoT devices are manufactured without taking security and privacy into consideration [6]. Thus, a large percentage of all devices, 20 billion connected IoT devices existed in 2020 according to Gartner’s reports, lack the proper defense mechanism that protects them from security attacks, which negatively impact the improvement and evolution of this technology [7].

The massive daily flow of the IoT connected devices introduces new threats and vulnerabilities that became hard to deal with. An example of such an issue occurred in October 2016, where simple IoT devices (cameras and DVRs) were exploited and converted to attack vectors in order to hack a big DNS provider (Dyn) [8]. This process led to a tremendous Internet outage and deactivated the availability of well-known websites, namely Amazon, Netflix, and Twitter. Another situation happened in the same year when Chinese researchers (a team of hackers) were able to control a car’s door locks, electronic features, and brakes causing a traffic accident [9].

These examples of network intrusion attacks not only disturb the availability of the device functions but also involve stealing important information and data about the device and the users utilizing it. Hence, it becomes essential to withstand these attacks. This creates a need for more intelligent mechanisms to connect and use IoT devices [10]. One of these mechanisms is Intrusion Detection Systems (IDS), which are a software application or tool that monitors the network to keep it secure and detect any malicious activity that takes place. Subsequently, the system reports and collects these violations by using event management and security information systems. Generally, different components could be part of any IDS, including the monitoring services that collect the data from the network, communication models that transfer the collected data to the IDS, and the classifiers that provide predictive models for different types of attacks. Based on the predictions’ results, different actions such as triggering alerts, excluding nodes from the networks, and many others might be taken. The communications among these components need to be authentic where proper authentication approaches [11,12] can be applied.

Given the computational and fundamental resource constraints, it is difficult to apply traditional security techniques to secure IoT devices directly. However, rule-based detection methods showed efficient outcomes [13,14,15]. Thus, anomaly-based detection mechanisms are crucial with the growth of IoT environments and technology. Generating big data from IoT devices can be of great benefit for machine-learning algorithms, where they can perform data analysis and deliver meaningful predictions and interpretations of the IoT devices. Thus, applying machine learning for IoT system security is considered an optimal opportunity to protect them from intrusion attacks, especially by detecting any outlier activity that emerges in the system. It is worth noting that machine learning also shows excellent performance in other areas such as [16,17,18,19,20].

Many popular datasets are introduced in the literature for intrusion detection, including ISCX2012 [21], UNSWNB15 [22] and CICIDS2017 [23]. Nevertheless, these datasets did not take into account the IoT environment when being collected. Various works started employing the intrusion detection benchmark using IoT environments, namely DS20S [24] and BoT-IoT [25]. However, these IoT datasets lack the novelty attack techniques that emerged in recent years and have an insufficient number of features. Therefore, modern datasets are presented to solve such problem like IoTID20 [26] and LITNET-2020 [27]. On one hand, the IoTID20 dataset was collected from different sources namely, laptops, smartphones, smart home devices, tablets, and so on. On the other hand, the LITNET-2020 dataset was gathered from attack network traffic of the KTU LITNET network. The two datasets are different in the environments they were generated from, where LITNET-2020 focuses on academic network traffic, while IoTID20 dataset focuses on home devices. Thus, in this work, we study and examine IoT intrusion detection for home device environments.

Another recent study examined the detection of IoT for webshell by utilizing ensemble techniques [28]. The work used several ensemble techniques in order to improve the detection criteria including random forest (RF), extremely randomized trees (ET), and voting. The results of their approach achieved excellent performance, particularly when using RF and ET for lightweight IoT cases and voting methods for heavyweight IoT scenarios.

Moreover, previous studies in the literature lack several points, such as considering modern datasets for intrusion detection and combining several techniques including different oversampling strategies, deep learning, and multi-layer approach, to produce high quality results. Therefore, in this work we introduce a multi-layer classification approach to detect and classify different types of intrusion by applying the following steps:

- Choosing a recent imbalanced dataset for IoT network applications called IoTID20. This dataset was extracted through monitoring a smart home and logging both normal and malicious behaviors. The malicious behavior is represented by four main threatening attacks, including Mirai malware, Denial of Service (DoS), Scan, and Man-in-the-Middle (MITM) attacks.
- Oversampling the imbalanced dataset using the Synthetic Minority Oversampling Technique (SMOTE) [29].
- Detecting intrusion activities from normal activities by applying a Single-hidden Layer Feed-forward Neural Network (SLFN).
- Identifying the four different types of intrusion activities including Mirai, DoS, Scan, and MITM attacks using a Deep Neural Network (DNN).

The main contributions of this paper fall into the following three points:

- Although the second step of the approach differentiates the intrusion activities from the normal ones, the oversampling technique is applied on the intrusion type identifier (ITI) label rather than the intrusion detector (ID) label, resulting in better oversampled data, thus generating better quality of results for the second step of the approach and eventually on the overall quality of the approach.
- The multi-layer construction of the approach allows better identification of the intrusion type, since the first and second stage of classification concentrate each on a single objective, allowing the learning process to depend on the correlation between related features only.
- The resulting predictive model from the proposed classification approach can then be injected by the IDS system based on the nature of the IoT application.

The rest of the paper is structured as follows. Section 2 summarizes the related works for intrusion detection of IoT networks. Section 3 presents the SMOTE oversampling technique, SLFN, the sequential model of DNN, and the Long Short-Term Memory (LSTM) techniques. Section 4 discusses the proposed deep multi-layer approach. Section 5 shows the experiments and results of the proposed approach. Finally, the last section concludes the work.

## 2. Related Works

As the field of IoT improves and spreads every day, the challenges related to its security and privacy have also evolved and increased [30,31,32,33,34,35]. Furthermore, the security threats exposing IoT devices have different impacts according to the domain that they operate in it, where each device may suffer from unique vulnerabilities accompanying the characteristics, procedures, and needs of that domain, including smart grids, industrial applications, smart homes, healthcare, and smart cities.

The security measure consists of several methods that aim to guarantee, preserve, and restore the safety of systems from malicious attacks. These attacks could come from several issues, including lack of compliance from the manufacturers about security standards, lack of user knowledge and awareness, problems in the update management process, lack of physical hardening, and botnet attacks. Therefore, having these issues with the IoT devices in the real-world environment increases the causes of exposure to intrusion threats. These threats could be, for instance, a Black Hole (dropping traffic in networks) [36], Storage Channel Attacks (transfer information by an unauthorized process) [37], Buffer Overflow (overruns the buffer boundary) [38], SQL Injection (code injection technique) [39] and so on [40].

The continuously increasing number of information leaks and attacks from IoT devices led to the speed up and increase in security and privacy research in the literature [41,42,43,44,45]. Even though several preventive measures are performed, IoT devices and systems are still exposed to malicious hackers and actors. Thus, the need for early detection of intrusion activities to reduce the negative impact of these devices has become more urgent than ever.

Fortunately, such threats can be diminished using detection measures, which are known as intrusion detection. One of the well-known intrusion detection methods that has gained attention recently in the literature is using machine learning techniques [46,47,48,49]. As a result, the standard intrusion detection techniques show improved quality by incorporating the machine learning techniques with them [50,51,52,53]. Machine learning techniques can be used to improve intrusion detection methods for traditional networks effectively.

Authors of [54], for example, stated that detection is an essential task to identify the anomaly in a dataset. They specified that intrusion detection is a very important area in the literature especially using techniques such as statistics and machine learning. Another work also mentioned the importance of applying intelligent tools to enhance standard intrusion detection [55]. In their work, the authors used an Optimum-Path Forest (OPF) classifier to detect intrusion in computer networks.

Therefore, the detection of intrusion attacks using machine learning techniques has become more effectual over the years. For example, Diro et al. [56] presented an intrusion detection system for Denial of Service (DoS) in IoT networks using deep learning. The proposed approach is compared with other traditional models using the NSL-KDD dataset and showed superior results. Furthermore, their approach is designed by taking into account two different schemes, namely, distributed detection and centralized. Consequently, the distributed attack detection scheme outperforms the centralized detection in terms of Accuracy. Another machine learning model for detecting IoT attacks is proposed by [57]. The suggested signature-based model utilized the artificial immune system to detect new attacks by self-learning and self-adaptation methods. However, due to the signature-based detection method, the model suffers from drawbacks even though it is combined with machine learning. Thus, without the proper combination of intrusion detection methods and machine learning, the performance could demonstrate shortcomings in certain cases.

In addition, a gradient boosted machine (GBM) was used for the intrusion detection problem [58]. This approach worked as a detection engine by applying the grid search to optimize the GBM parameters. The GBM-Grid model was evaluated on three different datasets, including UNSW-NB15, NSL-KDD, and GPRS. When compared with GAR forest, tree-based ensembles, and fuzzy classifiers, the proposed approach achieved better results in terms of Accuracy and specificity. Another similar work was performed by [59], where the Random forest-based Intrusion detection systems were investigated to study their performance in terms of false alarm rate and Accuracy. They employed GPRS, NSL-KDD, and UNSW-NB15 datasets for their research. The proposed approach is compared against several classifiers, namely the Multilayer perceptron, NBTree, and an ensemble of both Random tree, and Naive Bayes. The results showed that the Random forest-based Intrusion detection systems outperform the other classifiers.

Furthermore, the work in [60] examined the IoTID20 dataset using three various subsets with normal traffic including, Scan attack, DoS attack, and MITM. However, the authors did not investigate the intrusion activities of all attacks (DoS, scan, Mirai, and MITM attacks) which are combined in our work. Another work examined the same dataset to predict the existence of the intrusion using a multi-stage classification approach based on clustering with oversampling. However, it does not predict the type of intrusion but only detects the existence of the intrusion.

The work in [61] proposed an Imbalanced Generative Adversarial Network (IGAN) for an intrusion detection model. Their approach consists of three folds, a deep neural network, feature extraction, and IGAN. The feature vectors are constructed using a feed-forward neural network, the IGAN is responsible for generating new samples, and the deep neural network is applied for the detection phase. Meanwhile the work in [62] presented a continuous authentication biometrics behavior system for tackling false rejects and false accepts. The examination takes place using 16 biometric datasets. Furthermore, [63] presented an anomaly-based method for intrusion detection in an IoT environment. The authors detect cyber threats through an edge computing paradigm that is similar to data sources. More details of [61,62,63] as well as a comparison with the proposed approach can be found in Table 1. The table shows the methods applied by the works and the existence of the intrusion detection process, the IoT system, and the multi attacks. It also shows if the dataset is an imbalanced dataset and whether it is a recent dataset.

Applying machine learning techniques with the IoT intrusion detection methods is gaining more attention from researchers, as shown in the previously mentioned works and other research such as [64,65]. However, our work differs from these works by proposing a multi-layer classification approach consisting of the detection of the existence of the intrusion activities and the classification of several types of intrusion activities. In addition, using the oversampling technique on the ITI label while applying the resulting oversampled dataset on the ID label is not common. Moreover, as far as we know, the use of the very recent IoTID20 dataset [26] for detecting the normal activities and the different types of attacks has not yet been implemented by researchers.

## 3. Related Terminologies

This section presents the terminologies related to the work proposed in this study. It includes the SMOTE, SLFN, DNN, and LSTM techniques.

### 3.1. SMOTE Oversampling Technique

Imbalanced datasets are challenging for researchers and practitioners performing the classification tasks because classifying imbalanced datasets has the tendency of aggressively identifying minority labels as majority class. Consequently, this generates more false positives and degrades the quality of the classification task outcomes [66,67]. This gives researchers and practitioners a challenge of avoiding aggressive prediction of the majority class caused by the lack of instances available for the minority class.

Thus, several oversampling techniques have emerged and become an essential preparatory step of applying any classification task considering imbalanced datasets. One of the common and well-known oversampling techniques is referred to as SMOTE [29]. SMOTE synthetically creates new instances of the minority class to give richer information to the classification technique and thus enhances the quality of the prediction. SMOTE synthetically creates new samples considering the feature space of the instances rather than the instances themselves as a whole. This technique focuses on the features of the instances and the relationships between them to fulfil the minority class with additional instances [68].

### 3.2. Single-Hidden Layer Feed-Forward Neural Network (SLFN)

**Figure 1:**
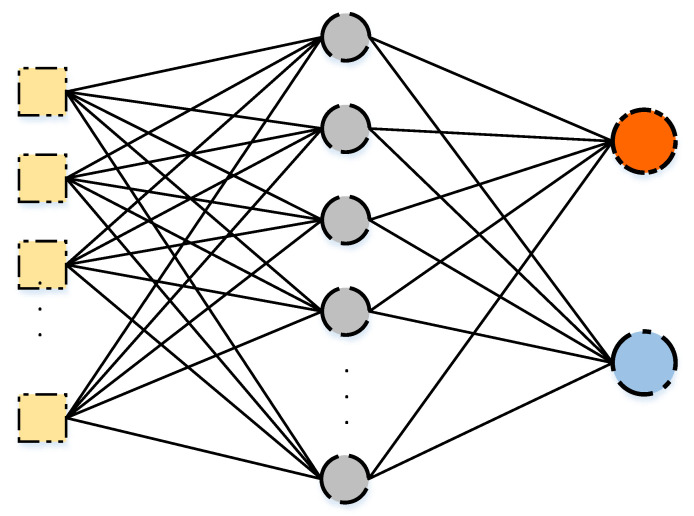
A Single Hidden Layer Feed-forward Neural Network.

An Artificial Neural Network (ANN) mimics the nervous system of a human [69]. The single hidden layer feed-forward artificial neural network is the simplest form of the ANN. The topology of the network is visible in Figure 1. The network consists of the following components:

- An input layer, a single hidden layer, and an output layer: The input layer holds the values of *N* features of the dataset. The hidden layer consists of *M* neurons, each holding the value generated by an activation function. The output layer holds the predicted outputs.
- Weights: Each node in the input layer is connected to each neuron of the hidden layer by a weight value, which is used for calculating the weighted sum of each neuron of the hidden layer. In addition, other weights connect each neuron of the hidden layer with each node of the output layer.
- Activation function: this function is responsible of generating the values of the neurons for the hidden layer.

The task of training the neural network aims at giving a high quality classification result by finding the relationships between the inputs and the outputs because the actual relationship in most cases cannot be recognized by traditional techniques [70]. This is done by tuning the weights and biases of the neural network to give better mapping, and thus increasing the Accuracy of the predicted labels, which is measured by a cost function.

The training process starts by calculating the weighted sum $WS_{j}$ of each neuron *j* of the hidden layer *J* by finding the sum of the product between the value $x_{i}$ of node *i* of the input layer and each weight $w_{ij}$ connecting the input nodes *i* and the neuron *j*. The result is then added to the bias $b_{J}$. This is observed by the following equation:

(1) $$WS_{j}=\sum_{i=1}^{N}w_{ij}x_{i}+b_{J}$$

The weighted sum $WS_{j}$ for each neuron *j* in *M* neurons is then passed to the activation function to generate an input value for the next layer representing the output layer.

A weighted sum $WS_{k}$ is also calculated for each node *k* of the output layer *K* by finding the sum of the product between the value $h_{j}$ generated by the activation function of the neuron *j* and each weight $w_{jk}$ connecting the output node *k* and the value $h_{j}$. The result is then added to the bias $b_{K}$. This is observed by the following equation:

(2) $$WS_{k}=\sum_{j=1}^{M}w_{jk}h_{j}+b_{K}$$

The weighted sum $WS_{k}$ represents the output obtained for each node *k* of the output layer.

### 3.3. Deep Neural Networks (DNN)

Deep neural networks consist of multiple hidden layers to train the data with multiple levels of abstraction [71]. They are based on the adjustments of the weights connecting the hidden layers to each other and to the input and output layers. As discussed in the previous section, the weights are used to compute the values for each node at each layer based on the values computed in the previous layer. In deep neural networks, the single hidden layer is extended to include several hidden layers and thus the computations are also extended, which gives better quality of predictions of the output layer generated from the network.

**Figure 2:**
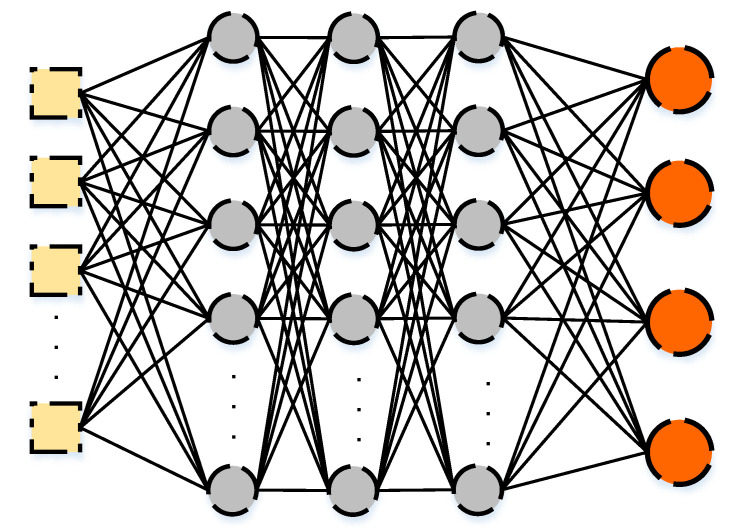
Sequential Model of a Deep Neural Network.

Figure 2 illustrates the existence of several layers and shows that the deep neural network includes extended computations with several hidden layers. Weights still exist between the input layer and the first hidden layer and between the last hidden layer and the output layer. The difference here is that there are additional weights connecting several other hidden layers. The same process is applied to each hidden layer, calculating a weighted sum and the activation function based on the input from the previous layer and the weights connecting it to the previous one. A general formula for calculating the weighted sum for the deep neural network is presented as follows:

(3) $$WS_{q}=\sum_{p=1}^{P}w_{pq}h_{p}+b_{Q}$$

where $WS_{q}$ is the weighted sum of neuron *q* of the hidden layer *Q*, *P* is the number of neurons of the previous layer, $w_{pq}$ is the weight connecting the neuron *p* of the previous hidden layer with the neuron *q* of the hidden layer *Q*, $h_{p}$ is the value of the neuron *p* of the previous hidden layer obtained by applying the activation function on the weighted sum of the neuron, and the bias $b_{Q}$ is the bias of the hidden layer *Q*.

The progress of applying deep learning approaches for several applications can be easily recognized in the literature. In addition, different models of the deep neural network can be found extensively [72]. The aforementioned process considering the sequential model (Seq) of a plain stack of layer is the most simple type of DNN. Further variations also exist including Autoencoders, Deep Belief Net, Convolutional deep Neural Networks (CNNs), Recurrent Neural Networks (RNN), Recursive Neural Networks, and Long Short-Term Memory (LSTM). The next section describes the LSTM deep neural network.

### 3.4. Long Short-Term Memory (LSTM)

LSTM was introduced by Sepp Hochreiter and Jürgen Schmidhuber [73] in 1997 and includes cells, input and output gates. Later, the forget gate and the peephole connections were added to LSTM by Felix Gers, Jürgen Schmidhuber, and Fred Cummins [74,75].

**Figure 3:**
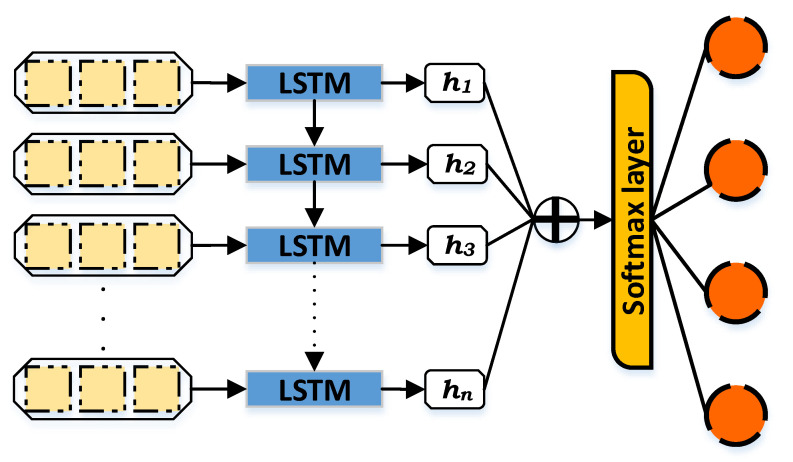
Long Short-Term Memory.

Figure 3 describes the structure of LSTM which is composed of an input layer, LSTM cell, hidden state, attention layer, softmax layer, and output layer. The LSTM cell contains the previously mentioned components (input, output, and forget gates as well as the cell layer). The cell is responsible for remembering the values at an exact time, while the gates are in charge of the input and output of the information flow. The output of the LSTM cell is known as the hidden state (*h*), it transfers the related information from the input items to the other objects. Furthermore, the attention layer or additive attention ⨁ in LSTM is intended to learn the significance of diverse information over time. Thus, it makes the distinctive information a higher priority over other information and eventually enhances the classification performance. On the other hand, the softmax layer or function works as a generalization process in order to normalize the output that comes from the network and enters output classes. The softmax normalizes the values by specifying decimal probabilities for each class ranging from zero to one, and can be calculated by the following equation:

(4) $$\sigma {\left(\vec{z}\right)}_{i}=\frac{e^{zi}}{\sum_{j=1}^{K}e^{zj}}$$

where $\vec{z}$ is the input vector, $z_{i}$ is the elements of the input vector. $e^{zj}$ denotes the standard exponential function which is applied on each element to add a small value if the input is negative and a large value if it is big, while $\sum_{j=1}^{K}e^{zj}$ is responsible for ensuring that the values remain between zero and one. Finally, *K* indicates the number of classes.

The LSTM is recognized as the most used type of RNN architecture. LSTM was introduced to solve the vanishing gradients problem of RNN by integrating non-linear controls to the RNN cell so that the gradient of the cost function does not vanish [76]. It is implemented to avoid the long-term dependency issue [77]. Therefore, remembering information for a long time is considered one of the main advantages of LSTM. With the existence of the unknown lags duration during the important events of learning, regular RNNs can not learn higher than 5–10 discrete-time of time presence lags; nevertheless, LSTM could learn more than 1000 timesteps using the constant error carousels [74]. Furthermore, the LSTM cell performs better due to its cell memory unit, where the cell vector can capsulize the previously-stored memory of the forgetting part, alongside adding a new portion of the information.

## 4. The Proposed Deep Multi-Layer Classification Approach

**Figure 4:**
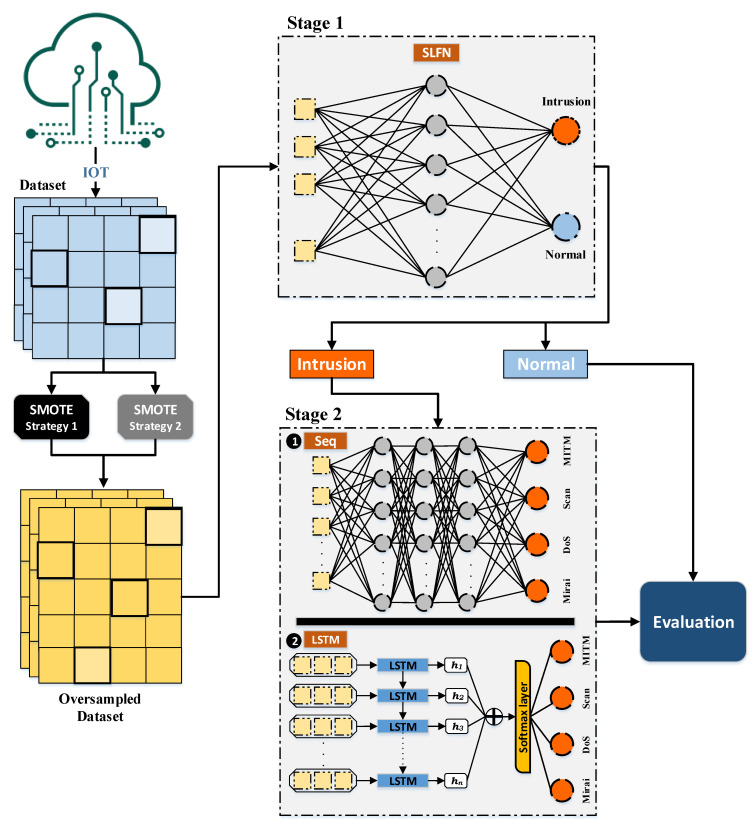
The proposed multi-layer classification approach.

A multi-layer classification approach is proposed in this paper to detect both the existence of an intrusion and the type of intrusion for IoT networks assuming an imbalanced type of datasets. The dataset is split to training instances and testing instances and then the proposed approach is applied. The proposed approach is illustrated by Figure 4. It starts by oversampling the training instances of the imbalanced dataset using the SMOTE oversampling technique to generate a more balanced dataset. It synthetically creates new training instances based on the correlation between the features. This produces more instances for the minority class to avoid aggressive prediction of the majority class. This is achieved by experimenting with two strategies; the first one is based on the ID label and the second one is based on the ITI label.

**Figure 5:**
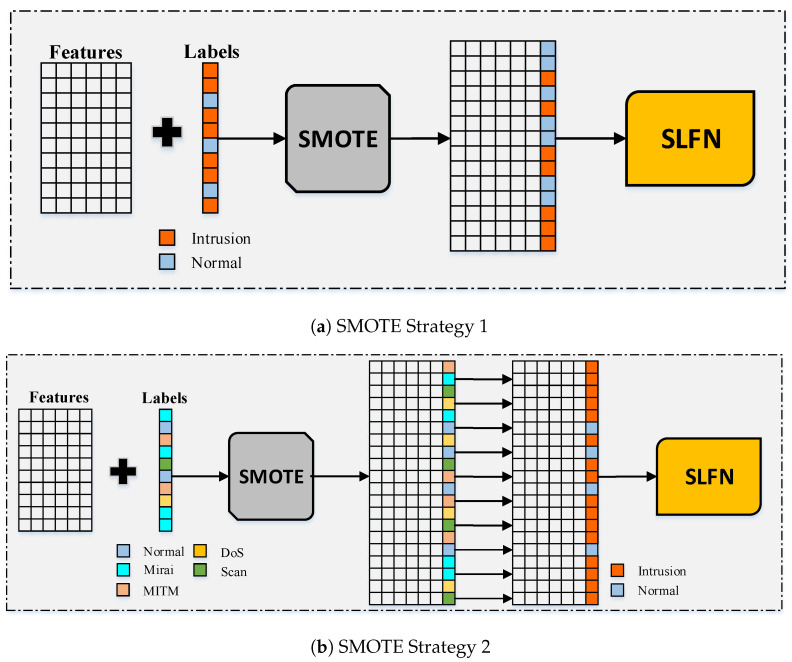
Adopted oversampling strategies in the first stage of the proposed approach.

The first strategy, which is illustrated in Figure 5a, starts by applying the SMOTE oversampling technique to the training instances of the dataset considering the ID label. This generates an enlarged dataset with more instances for the minority class which is the normal labeled instances according to the oversampling ratio specified. Figure 5a shows that the enlarged dataset has an equal number of the normal labels and the intrusion labels considering a ratio value of 1 for oversampling. The resulting enlarged dataset is then considered as an input to the SLFN classifier, which will be discussed briefly. The second strategy, which is illustrated in Figure 5b, differs from the first strategy as it applies the SMOTE oversampling technique on the training instances of the dataset considering the ITI label rather than the ID label. This generates an enlarged dataset with more instances for all the classes except for the majority class which is the Mirai intrusion type. It also differs from the first strategy in that the ITI labels are then converted to their corresponding ID labels. The enlarged dataset along with the generated ID labels are then considered as an input to the SLFN classifier.

The updated enlarged dataset generated from the SMOTE oversampling technique from Figure 4 follows a two layer classification stage as follows:

- Classification of ID label by the SLFN technique.
- Classification of the ITI label by the DNN technique.

The first stage considers the classification by the SLFN technique for detecting the ID label. The oversampled dataset generated by the SMOTE technique is considered a balanced dataset with intrusion and normal activities. The SLFN network, as discussed in Section 3.2, consists of an input layer, single hidden layer, and an output layer. The input layer consists of the features of the oversampled dataset and the output layer consists of two neurons representing the intrusion and normal labels. The network is responsible for finding the relations between the input features and the output labels by adjusting the weights of the network to reduce the error between the predicted values and the target values of the labels.

On the other hand, the second stage considers the classification by DNN, in which multiple hidden layers exist, by experimenting with both the sequential and the LSTM techniques. Both techniques classify the instances representing the intrusion activities only, which are generated from the first stage of classification, based on the ITI label, into different types of intrusion activities including Mirai, DoS, Scan, and MITM types. The sequential model consists of the input features layer, multiple hidden layers, and an output label layer of four neurons representing all the types of intrusion. In contrast, the LSTM model consists of input features layer, LSTM cell, hidden state, attention layer, softmax layer, and output label layer. The LSTM cell remembers the values producing the hidden state as an output to the LSTM cell. The additive layer learns the significance of the diversity of information and the softmax layer generalizes and normalizes the information. This produces the predictive values of all four types of intrusion.

The output to the two stages of classification is a two-layer classification model that is able to predict if an instance represents a normal activity or a Mirai, DoS, Scan, or MITM intrusion activity.

Finally, an evaluation process is performed on the predicted labels including the predicted normal activity label generated from the first stage of classification and the predicted type of intrusion label generated from the second stage of classification.

The time complexity of the proposed approach can be analyzed by the time complexity of the two stages of classification. The time complexity for the first stage of the classification by SLFN is $O(N_{epochs}*N_{patterns}*N_{in}*N_{out})$ [78] where $N_{epochs}$, $N_{patterns}$, $N_{in}$, $N_{out}$ represent the number of epochs, hidden neurons, input features, and output nodes, respectively, and the time complexity for the second stage of the classification by LSTM technique is O(W) where *W* represents the number of weights [79].

The advantages of using a multi-layer approach of classification compared to single classification approach include giving each classifier a more specialized classification task which results in better overall classification. In addition, the results obtained from the SLFN classifier are given to the DNN classifier where normal activities are not interfered with by those of intrusion type, and thus allowing the DNN to give a better weighting process for the features that are directly related to the type of intrusion rather than both the existence of intrusion and the type of the intrusion. At another level, although the first stage of classification considers detecting intrusion activities from normal ones, the oversampling task considers the ITI label rather than the ID label allowing better representation of the oversampled dataset.

Several challenges exist for applying the proposed multi-layer approach. The challenges are overcome by applying several experiments with different settings to address the best settings’ values for the dataset studied in this paper, which is discussed in Section 5. These challenges include the following:

- Selecting the oversampling ratio for balancing the imbalanced dataset.
- Selecting the number of neurons for the layers of the SLFN and the DNN.
- Selecting the number of layers for the DNN.
- Generating high quality results of the classification process for both4 ID label and ITI label, which are represented by the ID label and the Mirai, DoS, Scan, and MITM ITI labels.

## 5. Experiments and Results

This section discusses the experiments conducted on the IoTID20 dataset and the results achieved from running the multi-layer approach. This section includes the environmental settings, the dataset description, the evaluation measures, the effect of the SMOTE oversampling technique on the data when classified by SLFN, the Intrusion detection of conducting the experiments using the proposed approach, a comparison between the proposed approach and other standard algorithms, and a summary of the discussion.

### 5.1. Environmental Settings

The experiments were run on a personal computer with an Intel core i7-1065G7 CPU and 1.30 GHz/16 GB RAM using Python 3.8. The imbalanced-learn [80], Scikit Learn [81], keras [82] Python libraries were used to run the SLFN, SMOTE, Sequential model, and LSTM techniques and to evaluate the results.

A different number of neurons were used for SLFN which were 50, 100, 150, and 200. An oversampling ratio values of 0.2, 0.4, 0.6, 0.8, and 1 were used for running the SMOTE oversampling technique on the ID label. Values of 50, 100, and 150 neurons with 2–3 layers were also tested with both the sequential model and LSTM.

### 5.2. Dataset

The IoTID20 dataset [26] is a recent intrusion activity detection dataset for IoT networks. It was generated in a smart home environment with a Wi-Fi router connected to victim devices which are the SKT NGU and EZVIZ camera and attacking devices which are the laptops, tablets, and smartphones.

The dataset consists of 80 features, 625,783 instances, and three types of labels. The dataset has a large number of features including a large number of flow-based features. It has both intrusion and normal activities, where the intrusion activities themselves are categorized into DoS, Mirai, MITM, and Scan attacks, which are further categorized to different other sub categories.

In this work, we considered two types of labels for the IoTID20 dataset which are the ID label indicating intrusion activities from normal ones and the ITI label indicating different types of intrusion. The distribution of the instances across the labels are observed by Table 2. It can be deduced from the table that the number of intrusion activities is approximately 15 times as much as the normal activities. It is also observed from the table that the Mirai type of intrusion is also a dominant type compared to the other types of intrusion activities which reflects an imbalanced dataset.

### 5.3. Evaluation Measures

The proposed approach is evaluated using Accuracy, Recall, and G-mean measures. These measures are based on the true positives (TP), true negatives (TN), false positives (FP), and false negatives (FN) values. The TP, TN, FP, and FN values reflect the number of right predictions of the positive class, the number of right predictions of the negative class, the number of wrong predictions of the positive class, and the number of wrong predictions of the negative class, respectively. The Accuracy, Recall, and G-mean measures are described as follows:

- Accuracy (ACC): It represents the correct predicted instances of all the classes relative to the number of instances. The Accuracy is calculated for the ID label by Equation (5) and for the ITI label by Equation (6).
- Recall (REC): It represents the ability to predict the right labels of a class as a ratio between the true prediction of the class and the correct labels of the class. The Recall is calculated for each class of the ID label and the ITI label by Equation (7).
- G-mean: It considers the Recall of all the classes. The G-mean is calculated for the ID label by Equation (8) and for the ITI label by Equation (9).
  (5) $$ACC_{ID}=\frac{TP_{Normal}+TP_{Intrusion}}{\#instances}$$
  (6) $$ACC_{ITI}=\frac{TP_{Normal}+TP_{DOS}+TP_{MITM}+TP_{Mirai}+TP_{Scan}}{\#instances}$$
  (7) $$REC_{class}=\frac{TP_{class}}{TP_{class}+FN_{class}}$$
  (8) $$G-mean_{ID}=\sqrt{REC_{\mathrm{Normal}}\times REC_{\mathrm{Intrusion}}}$$
  (9) $$G-mean_{ITI}=\sqrt[{5}]{REC_{\mathrm{Normal}}\times REC_{\mathrm{DOS}}\times REC_{\mathrm{MITM}}\times REC_{\mathrm{Mirai}}\times REC_{\mathrm{Scan}}}$$

### 5.4. Effect of Oversampling Technique on Intrusion Classification

The aim of this section is to investigate the effect of the SMOTE oversampling technique on the quality of the outcomes achieved by the first stage of the approach. First, a different number of neurons was used on SLFN. The aim was to find the number of neurons generating the best quality of output for the IoTID20 dataset. Second, two different oversampling strategies were used on SLFN with the best number of neurons achieved from the first part of the experiments.

Table 3 shows the results obtained from running the SLFN with a different number of neurons including 50, 100, 150, and 200 neurons in terms of Accuracy, Recall, and G-mean measures. It can be observed from the table that SLFN with 150 neurons has the best values for the Recall of the intrusion label and the G-mean. SLFN with 200 neurons shows the best value for Accuracy by sacrificing the Recall of the intrusion to the Recall of the normal, and causing a decrease in the G-mean value, which is critical for IoT networks. Furthermore, having a high value for the Recall of the normal label for SLFN with 50 neurons causes the same decrease in the Recall of the intrusion and the G-mean. Thus, we consider the SLFN with 150 neurons the best option for the second part of the experiments.

**Figure 6:**
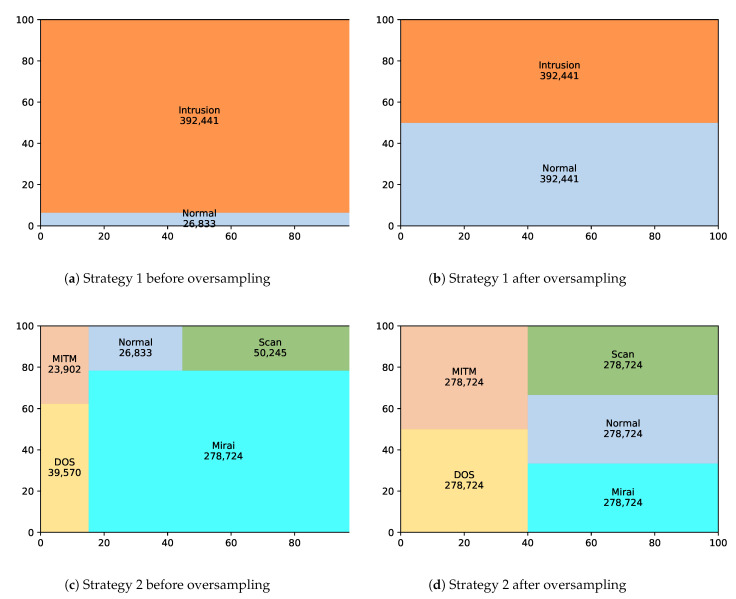
The distribution of the training instances by applying Strategy 1 and 2 of the SMOTE oversampling technique.

The second part of the experiments considered the two strategies, discussed in Section 4, for oversampling the dataset and classifying it using SLFN. The distribution of the training instances of the dataset by applying both strategies is illustrated by Figure 6. Figure 6a,b show the effect of strategy 1 on the training instances of the dataset before applying the oversampling technique and after applying it, respectively. The imbalanced training instances in the first figure show that the number of the instances labeled as intrusion has a larger value than the number of instances labeled as normal, but after applying the oversampling technique with an oversampling ratio of 1, the number of instances of the normal label increases and becomes equal to the number of instances for the intrusion label, causing an enlarged training dataset. In contrast, Figure 6c,d show the effect of strategy 2 on the training instances of the dataset before applying the oversampling technique and after applying it, respectively. In this strategy, the number of instances of the ITI label is the one considered, which includes those for the normal label. It is observed from the first figure that the number of instances for the Mirai intrusion type has a larger value than the number of instances for the other types. After applying the oversampling technique on the ITI label, the number of instances for all the labels increases and becomes equal to the number of instances for the Mirai intrusion label, causing an enlarged training dataset.

**Figure 7:**
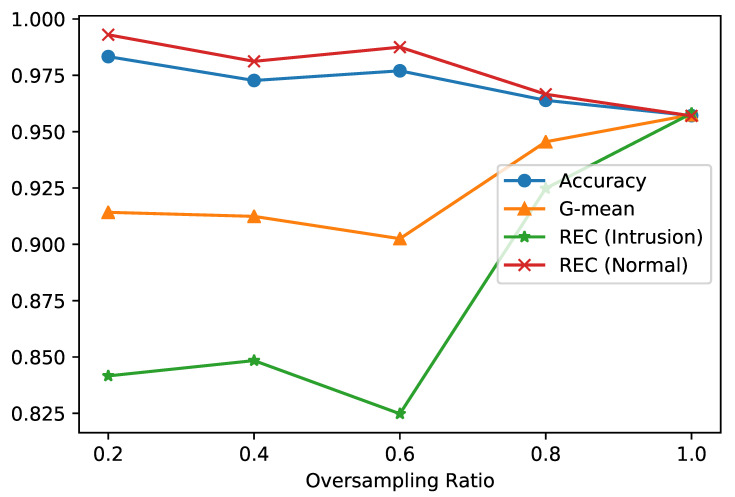
Evaluation of different oversampling ratio values for strategy 1.

Both strategies were tested on the IoTID20 dataset to find the best strategy for the approach proposed. The first strategy was tested using different values of the oversampling ratio values which are 0.2, 0.4, 0.6, 0.8, and 1. Figure 7 shows the results of applying the first strategy with the different values of the oversampling ratio for Accuracy, Recall, and G-mean measures. It is observed from the figure that the oversampling ratio of one is the best for G-mean and the Recall of the intrusion label, which is our main focus for IoT networks. Thus, we consider it for comparison with strategy 2.

Table 4 shows the results of applying both strategies. Strategy 2 was tested using the auto value for the oversampling ratio as we did not consider enlarging the number of instances for one label, which is the normal in the case of strategy 1, but rather we considered enlarging the number of instances of several labels which include the normal, MITM, DOS, and Scan labels excluding the instances of the majority label which is the Mirai label. Thus, the auto ratio, which oversamples all classes except the majority class, is considered for oversampling the multi-label instances by equalizing the number of instances for all the labels. It is observed from the table that the second strategy recognizably outperforms the first one for all the evaluation measures. The values of Accuracy, Recall, and G-mean are approximately touching the optimal value of one for strategy 2, which indicates an outstanding performance for the proposed strategy. The reason behind this outstanding behavior is that the oversampling technique recognizes more detailed information about the training instances by considering the ITI label for oversampling the training instances, and thus giving more representative training instances for the SLFN classification technique, which results in a better detection of the intrusion activities by the SLFN classification technique.

### 5.5. Intrusion Type Detection Using Deep Learning

The proposed multi-layer approach is applied considering two deep learning algorithms which are the Sequential model and LSTM with different settings. Settings include 2 or 3 layers for each and 50, 100, 150, and 200 neurons at each layer. As discussed earlier, strategy 2 of the SMOTE oversampling technique is selected which considers the ITI label and the SLFN technique with 150 neurons. The aim is to find the best setting for the deep learning stage with the best quality of output for the IoTID20 dataset. Table 5 shows the results of applying the approach for the aforementioned settings. The Recall value of the normal instances for all the techniques are the same since the first stage of the approach is applied for all these techniques in advance which predicts the normal label. Different techniques achieved the best results for the Recall for different types of intrusion activities. However, a two-layer LSTM with 150 neurons (SMOTE-SLFN_(150)_-LSTM_(150,150)_) has the best value for Accuracy and G-mean, which indicates a competitive classification results for such an imbalanced dataset.

### 5.6. Comparison with Standard Algorithms

The proposed multi-layer approach is compared with other well-known single classification algorithms including the sequential model, LSTM, SLFN, Naive Bayes (NB), and k-nearest neighbor (KNN).

Table 6 shows the values of the evaluation measures and the standard deviation for the different algorithms. The standard deviation is not shown for KNN and NB algorithms since they produce the same results if tested by several runs. It is observed from the table that the Recall for the Scan and the DoS intrusion types are not accurately predicted by most of the classification algorithms. In addition, the proposed approach has the best Recall value for the Scan intrusion type and acceptable Recall values for DoS, MITM and Mirai intrusion types. In the case of normal behaviour, the proposed approach achieved the highest Recall value in comparison with the other standard algorithms. The Accuracy achieved by the proposed approach is close to the Accuracy value of the KNN, which has the best Accuracy value. Further, KNN also shows better Recall for the Mirai and MITM intrusion types but does not outperform the proposed approach for the Recall of Normal, DOS, and Scan intrusion types. On the other hand, Accuracy and Recall values can not give good insights for the quality of the predictions of imbalanced datasets. The proposed approach outperforms the other techniques in terms of G-mean, which is the main target of this study, since the dataset is imbalanced and we need to make sure that the classification approach gives quality prediction to both the majority class and the other classes. Finally, the standard deviation values for the proposed approach, the sequential model, LSTM, and SLFN are acceptable for all and reflect stable approaches.

### 5.7. Summary and Discussion

The proposed approach includes a deep multi-layer classification for IoT datasets that are oversampled using the SMOTE technique. The approach is shown to overcome some challenges using different settings which include the following:

- Different strategies for the SMOTE technique.
- A different number of neurons for the SLFN.
- A different number of layers and number of neurons for the DNN.
- Different types of DNNs including a Sequential model and LSTM.

According to the experiments, the best settings achieved include oversampling by the ITI label, 150 neurons for SLFN, and 2 layers and 150 neurons for LSTM.

A multi-layer approach allows the classifiers at each layer to give more special attention to their specialized task including the detection of intrusion by the SLFN technique and the identification of the intrusion type by the LSTM technique. It also allows LSTM to give better weighting representation of the features since the normal instances are already detected by the SLFN. In addition, oversampling by the ITI label but classification by the ID label is not trivial but, according to experiments, applying this strategy gives better representation of the oversampled dataset, which results in better quality of the classification task.

## 6. Conclusions and Future Works

In this paper, a deep multi-layer classification approach is proposed consisting of the following components: an oversampling technique using SMOTE to solve the issue of imbalance datasets. Two strategies are proposed to enhance the classification results. The first stage of classification by the SLFN technique predicts the intrusion and normal activities. The second stage of classification by DNN predicts the type of intrusion activities.

The experiments are conducted on a recent dataset named IoTID20, which shows that the proposed approach outperforms the other approaches by predicting the existence of the intrusion activities and the type of intrusion. The results show that the proposed approach has the highest values for the Recall for the Normal activities and the Scan intrusion type, and an acceptable Recall values for the other intrusion types, when comparing it with the other algorithms. It also has the highest G-mean value, which is an important indicator for the high quality of the proposed approach, since the IoTID20 dataset is an imbalanced dataset, This ensures that the prediction is not biased toward the majority class. The early and accurate detection of the IoT attacks has a significant impact on the IoT networks’ performance and their applications.

A limitation of the experiments is the consideration of the IoTID20 dataset apart from other different kinds of datasets. The reason is that the dataset covers large variations of normal and intrusion activities and a large number of different features. In addition, the availability of the intrusion type identifier label along with the intrusion detector label for the dataset, which is considered in the study, makes the dataset distinguished over many other common datasets. Another limitation is the exclusion of the intrusion sub type identification label, which can be studied in future works. In addition, more experiments are to be expected for the IoTID20 dataset where a comparison can be made between the future works and this work.

For future work, the work can be extended to provide a more detailed prediction for the sub categories of the intrusion by applying another layer to the proposed approach. In addition, evolutionary computation techniques can be added to the proposed multi-layer approach to explore more enhanced prediction of the type of the intrusion activities. Moreover, different machine learning-based intrusion systems can be deeply analyzed and compared to study (a) the impact of delaying the detection and exclusion of different types of attacks on the services provided by IoT networks (b) the complexity of their prediction systems and the corresponding resource consumption, especially in case of IoT devices with limited resources. Finally, the proposed classifier could detect the attacks considered to generate the dataset, including flooding, spoofing, and scanning attacks. However, other attacks could be considered in future research, such as impersonation, Sybil, and other DoS attacks.

## Figures and Tables

**Table 1:** A comparison between the proposed approach and other methods in the literature.

Publication	Method	Intrusion Detection	IoT	Multi-Attacks	Imbalanced	Recent Dataset
[61]	Deep learning	Yes	No	Yes	Yes	No
[62]	Behavioral Biometrics	No	No	Yes	No	No
[63]	iForest and LOF	Yes	Yes	Yes	No	Yes
Proposed	Deep learning	Yes	Yes	Yes	Yes	Yes

**Table 2:** IoTID20 Dataset instances including the ID label and ITI label.

ID Label		ITI Label	
Normal	40073	Normal	40073
Intrusion	585710	DoS	59391
		Mirai	415677
		MITM	35377
		Scan	75265

**Table 3:** Performing SLFN on a different number of neurons. Bold font indicates best result.

Technique	ACC	REC_Intrusion_	REC_Normal_	G-Mean
SLFN_(50)_	0.9847	0.7736	**0.9992**	0.8792
SLFN_(100)_	0.9841	0.8008	0.9967	0.8934
SLFN_(150)_	0.9847	**0.8066**	0.9969	**0.8967**
SLFN_(200)_	**0.9853**	0.7895	0.9987	0.8880

**Table 4:** Oversampling by SMOTE for SLFN of 150 neurons on different values of sampling strategy. Bold font indicates best result.

Strategy	ACC	REC_Intrusion_	REC_Normal_	G-Mean
1	0.95709	0.95808	0.95702	0.95755
2	**0.99981**	**0.99803**	**0.99999**	**0.99901**

**Table 5:** Intrusion type detection using a deep learning sequential model and LSTM with a different number of layers and neurons. Bold font indicates best result.

Technique	ACC	REC_Normal_	REC_DOS_	REC_MITM_	REC_Mirai_	REC_Scan_	G-Mean
SMOTE-SLFN_(150)_-Seq_(50,50)_	0.7714	0.9985	0.1832	0.6454	0.6870	0.9857	0.6033
SMOTE-SLFN_(150)_-Seq_(100,100)_	0.7323	0.9985	0.0045	0.6265	0.4828	0.9873	0.2668
SMOTE-SLFN_(150)_-Seq_(150,150)_	0.6446	0.9985	0.1457	0.5142	0.8455	0.6906	0.5346
SMOTE-SLFN_(150)_-Seq_(200,200)_	0.7277	0.9985	0.3303	0.5971	0.4881	0.9613	0.6211
SMOTE-SLFN_(150)_-Seq_(50,50,50)_	0.6973	0.9985	0.0001	0.5748	0.5354	0.9828	0.1214
SMOTE-SLFN_(150)_-Seq_(100,100,100)_	0.5451	0.9985	0.0002	0.3719	0.4571	**0.9989**	0.1242
SMOTE-SLFN_(150)_-Seq_(150,150,150)_	0.7611	0.9985	0.5116	0.6526	**0.9249**	0.5928	0.7119
SMOTE-SLFN_(150)_-Seq_(200,200,200)_	0.7746	0.9985	0.5435	0.6525	0.4236	0.9445	0.6765
SMOTE-SLFN_(150)_-LSTM_(50,50)_	0.7938	0.9985	0.4511	0.6489	0.8224	0.9392	0.7425
SMOTE-SLFN_(150)_-LSTM_(100,100)_	0.8460	0.9985	0.4845	0.7261	0.8188	0.8926	0.7619
SMOTE-SLFN_(150)_-LSTM_(150,150)_	**0.8620**	0.9985	**0.5558**	0.7476	0.8334	0.8540	**0.7835**
SMOTE-SLFN_(150)_-LSTM_(200,200)_	0.7596	0.9985	0.1058	0.6435	0.5971	0.9907	0.5259
SMOTE-SLFN_(150)_-LSTM_(50,50,50)_	0.8338	0.9985	0.5356	0.7034	0.8403	0.8913	0.7762
SMOTE-SLFN_(150)_-LSTM_(100,100,100)_	0.8163	0.9985	0.5557	0.6696	0.8375	0.9373	0.7816
SMOTE-SLFN_(150)_-LSTM_(150,150,150)_	0.8538	0.9985	0.4866	**0.7551**	0.9026	0.7468	0.7562
SMOTE-SLFN_(150)_-LSTM_(200,200,200)_	0.7208	0.9985	0.2476	0.5659	0.7784	0.9650	0.6373

**Table 6:** Comparative experiments of the proposed approach with other well-known classification techniques. Bold font indicates best result.

Technique	Accuracy	REC_Normal_	REC_DOS_	REC_MITM_	REC_Mirai_	REC_Scan_	G-Mean
SMOTE-SLFN_(150)_-LSTM_(150,150)_	0.8620 ± 0.0260	**0.9985** ± 0.0002	0.5558 ± 0.0570	0.7476 ± 0.0303	0.8334 ± 0.0873	**0.8540** ± 0.0788	**0.7835** ± 0.0247
Seq_(150,150)_	0.7724 ± 0.0054	0.9956 ± 0.0017	0 ± 0	**0.9940** ± 0.0024	0.2752 ± 0.0899	0 ± 0	0 ± 0
LSTM_(150,150)_	0.7945 ± 0.0048	0.9931 ± 0.0039	0.0010 ± 0.0836	0.9933 ± 0.0587	0.6298 ± 0.1281	0.0003 ± 0.2973	0.0005 ± 0.1010
SLFN_(150)_	0.8162 ± 0.0086	0.9978 ± 0.0002	0.1578 ± 0.0474	0.9747 ± 0.0071	0.7845 ± 0.1123	0.1231 ± 0.0417	0.4307 ± 0.1802
NB	0.5653	0.9877	**0.9606**	0.5510	0.7714	0.0139	0.3547
KNN	**0.8648**	0.9973	0.5205	0.9251	**0.8883**	0.5769	0.7555

## Data Availability

Data available in a publicly accessible repository at https://sites.google.com/view/iot-network-intrusion-dataset/home (accessed on 21 April 2021). The data presented in this study are openly available at https://doi.org/10.1007/978-3-030-47358-7_52 (accessed on 21 April 2021) [26].
